# CORESS feedback

**DOI:** 10.1308/003588412X13171221591097

**Published:** 2012-05

## Abstract

This edition of CORESS feedback reinforces the very basic principles of obtaining and using an accurate history and examination to make an appropriate diagnosis in the face of equivocal or uninformative investigations and failing equipment. Case 126 illustrates once again the potential deleterious consequences of failing to check a drug correctly prior to administration.

We are grateful to the clinicians who have provided the material for these reports. The online reporting form is on our website (www.coress.org.uk), which also includes all previous feedback reports. Published contributions will be acknowledged by a ‘Certificate of Contribution’, which may be included in the contributor’s record of continuing professional development.

CORESS relies heavily on the expertise of the specialty members of the Advisory Board in the preparation of feedback reports and dissemination of safety information related to surgical practice. The organisation is grateful to the following members of the Advisory Board and Board of Directors who have contributed to published reports in 2010 and 2011:

Board of Directors: Viscount Bridgeman, Mr Chris Chilton, Mr Martin Else, Professor Nicholas Gair, Mr Adam Lewis CVO, Miss Clare Marx, Mr Andrew May, Lord Bernard Ribeiro, Mr Frank Smith, Mr Peter Tait, Mr Denis Wilkins.

Advisory Board: Ms E Baird, Mr Daryl I Baker, Mr Ken Catchpole, Dr Lauren Morgan, Mr Stephen Clark, Mr Robert Davies, Mr Mark Deakin, Ms D Eastwood, Mr Barry Ferris, Mr Mark Fordham, Mr Paul J Gibbs, Mr Grey Giddins, Mr Robert Greatorex, Mr Mervyn Griffiths, Mr John Hammond, Mr William Harkness, Mr M Hemadri, Mr Richard Holdsworth, Miss Claire Hopkins, Professor Zygmunt Krukowski, Mr N Mamode, Mr Ian Martin, Surgeon Commander Mark Midwinter, Mr J Richard Novell, Professor Gerald O’Sullivan, Dr Gerard Panting, Mr Mike Pittam, Dr Mike Powers QC, Ms Patricia Scott, Professor Alastair Thompson, Dr J P van Besouw, Mr Mark Vipond, Mr David Webster, Mr Michael Wyatt.

## Plate or no plate? (Ref 108)

A 55-year-old man presented to the accident and emergency department (A&E) having swallowed his own partial denture one hour previously. He complained of severe throat pain and a foreign body sensation in the throat. This was followed, 50 minutes later, by intense pains throughout his chest. There was no dyspnoea or dysphonia. Initial examination of the oral cavity was unremarkable. X-rays of the lateral soft tissues of the neck and chest failed to demonstrate any abnormality or foreign body. The patient was reassured and requested to re-attend if there were no improvement in symptoms within 72 hours. He re-attended A&E 48 hours later complaining of persistent sore throat, dysphagia to solids and odynophagia. Neck and oral cavity examinations were unremarkable. The patient was again reassured and an ENT outpatient appointment was arranged as follow-up.

He attended this five weeks later still complaining of a sore throat. Indirect laryngoscopy demonstrated candida infection of the posterior pharyngeal wall but no other abnormality or foreign body. The original plain films were reviewed and no foreign body seen. The patient was reassured and prescribed anti-fungal lozenges. He re-attended A&E one week after the ENT outpatient appointment with persistent sore throat, odynophagia and dysphagia to solids. This time, flexible nasolaryngoscopy was performed, which demonstrated inflammation of laryngeal mucosa but no pooling of saliva. Neck examination failed to elicit surgical emphysema. He was given a week’s course of oral antibiotics, analgesia and a further outpatient appointment at which, despite complaining of a persistent sore throat, neck examination was normal again. He was reassured and discharged.

Eight months after the original incident he was re-referred by his general practitioner with dysphagia to solids and sore throat. He had dysphonia. Fibreoptic examination demonstrated left vocal cord palsy. Neck examination elicited left-sided tenderness but no other abnormality. A contrast CT scan of the neck and chest demonstrated a double lumen of the oesophagus extending from the level of the cricoid down to the sub-cervical oesophagus with what appeared to be a blind ending pouch superiorly. This was causing a mass effect in the left side of the neck. No foreign bodies were seen. Pharyngoscopy and oesophagos- copy were performed and a large mass was seen arising from the left postero-lateral wall of the oesophagus with a smooth mucosal cover. The oesophagoscope was unable to pass beyond the mass. No foreign body was seen. Biopsies were taken and reported as showing inflammation but no malignancy. An open surgical exploration of the left side of the neck was therefore undertaken. At operation the partial denture was found lying in the left tracheal-oesophageal groove. Post-operatively the patient was fed by nasogastric tube for five days and apart from the persistent left vocal cord palsy, made an uncomplicated recovery.

### Reporter’s comments

Clinicians placed undue emphasis on negative investigations at the expense of the history and delay in appropriate referral to a specialist team occurred. This was compounded by a lack of knowledge of the potential radio transparency of denture material.

### CORESS comments

The primary lesson is to listen to the patient’s history. The initial presentation of swallowed partial denture, sore throat, and severe chest pain should, with hindsight, have elicited a more effective specialist response, probably including early computed tomography scan. It appears to be well known in dental circles that dental plates are often translucent on x-ray. This knowledge needs to be disseminated to A&E and to ENT clinics. A&E doctors should have a high index of suspicion with ingested foreign bodies and a low threshold for referring patients with suspected sharp foreign body ingestion to the appropriate specialty for further assessment.

## An appendix too far (Ref 112)

An 18-year-old male with right iliac fossa pain was diagnosed with acute appendicitis and underwent a lengthy laparoscopic appendicectomy. The operation was undertaken by a trainee with his consultant supervising but unscrubbed. Post-operatively the patient failed to improve clinically and developed fluctuating pyrexia. Ultrasound suggested the presence of a pelvic abscess and the patient was taken back to theatre for laparoscopic drainage. At laparoscopy, a mass was found in the right iliac fossa, which proved very difficult to dissect. The procedure was converted to a midline laparotomy. The operative findings were surprising. There was an indurated mass and inflamed ileocaecal thickening with a tubular structure at the junction which could only have been the appendix. On this occasion appendicectomy was undertaken successfully.

Subsequent pathological examination of the previously resected ‘appendix’ revealed a piece of mesenteric fat that had been mistaken for the appendix by the trainee and his mentor.

### Reporter’s comments

The operation was complicated by a difficult dissection and obvious failure to identify the appendix correctly. Early conversion would have been appropriate. From his unscrubbed position, the supervisor failed to recognise the trainee’s mistake in tissue identification.

### CORESS comments

In a difficult laparoscopic appendicectomy, early conversion should be considered by the surgeon, inexperienced or otherwise. The unscrubbed supervisor has responsibility for the procedure and should either have scrubbed for the procedure or supervised the trainee more closely. The Advisory Board suggested that an escalating series of steps should occur in this situation: step 1 - supervisor scrubs in (increases his or her situational, tactile and perceptual awareness); step 2 - supervisor takes control of procedure; step 5 - conversion to open procedure as necessary.

## Kit conundrum (Ref 113)

An elderly lady with a history of hypertension was taken to theatre for reduction of a dislocated hip prosthesis. On arrival in the anaesthetic room her systolic blood pressure was recorded as 200mmHg. Despite receiving intravenous hydralazine her blood pressure failed to reduce to a satisfactory level. She was returned to the ward where she received 10mg of amlodipine, orally. Her systolic blood pressure gradually dropped to 150mmHg and she was later returned to theatre. Induction of anaesthesia was performed with propofol and maintained with sevofiurane, with the aim of further reducing blood pressure. However, when she was taken from the anaesthetic room into theatre her systolic blood pressure measured by automated sphygmomanometer was now 60mmHg.

Despite repeated boluses of metaraminol, systolic blood pressure measurements remained at 60mmHg. On feeling a radial pulse and observing good peripheral perfusion, the anaesthetist changed the blood pressure cuff to the opposite arm but the recorded pressure was still low and the patient was given a further bolus of metaraminol. A new blood pressure cuff was eventually sourced and with this her systolic blood pressure was recorded at 240mmHg. The patient was sent back to recovery until her blood pressure normalised and then returned to theatre, finally undergoing successful reduction of her hip.

### Reporter’s and CORESS comments

This was an incident related to equipment malfunction. Equipment should always be carefully checked preopera- tively and for essential kit, backup should be available. The temptation to rely on erroneous instrument readings in the face of contradictory clinical parameters may lead to inappropriate decisions with deleterious consequences. Where a discrepancy exists, early equipment checks may highlight the problem. Although profound hypotension warrants an immediate response this should only be acted on after full assessment of the patient’s clinical condition and haemody- namic status.

## Wrong dose heparin (Ref 126)

There are times when the vascular surgeon becomes worried about systemic coagulopathy. On this occasion I was performing a straightforward carotid endarterectomy, having stopped clopidogrel seven days before surgery but having continued the patient on aspirin 75mg daily. Before cross-clamping the carotid artery I requested the usual 5,000 units of heparin to be given intravenously.

The endarterectomy was uneventful but in recovery it became apparent that the skin edges were bruising; the Re- divac drain filled with blood and the tissues became inspissated with haematoma. This appeared to be due to a coagulopathy and we immediately ordered blood tests to check internationalised normal ratio, activated partial thromboplastin time (APPT), platelet count and haemoglobin. The APPT was greater than 2.5 and this was therefore reversed judiciously with small incremental doses of protamine. Protamine is a drug I dislike because of its hypotensive properties and indeed the systolic blood pressure fell to 40mmHg for a short period. The situation resolved without further adverse sequelae but clearly both hypotension and use of protamine could have resulted in adverse thrombotic consequences for the freshly endarterectomised site.

The cause of this problem was inadvertent intravenous injection of 25,000 units of heparin rather than the 5,000 units requested. We usually use ampoules of 1,000 units per ml but on this occasion the concentration, given by mistake, was 5,000 units in 1ml.

### CORESS and reporter’s comments

Clearly this problem was due to human error but the question of whether hospital operating theatres should stock only one concentration of heparin rather than both, or whether they should be stored separately, is raised. The problem has already been brought to the attention of Sir Liam Donaldson by Professor Brian Toft.^1^ The article^1^ was sent to the Chief Medical Office, who responded: ‘the NPSA guidance on anticoagulants recommends the use of 1,000 units per ml heparin products in clinical areas and the subsequent removal of higher strength products such as those involved in the incident you are investigating’. Clearly this message needs to be disseminated more widely.

## ...and finally

The Safe Anaesthesia Liaison Croup (SALG) of the Royal College of Anaesthetists notes that incidents of wrong- site local anaesthetic block before surgery continue to be reported (http://www.rcoa.ac.uk/docs/SALG-Statement- WSB.pdf, cited April 2012). Following a trigger incident, the National Reporting and Learning Service identified 67 further reports of wrong-site block in a period of 15 months. The majority of blocks were administered by anaesthetists.

Some reports gave reasons as to why this occurred, including distraction of the anaesthetist, lack of mark specific to the block, lack of anaesthetic time-out, and discrepancy between the operating list and mark or consent form.

Some reports stated that the site was marked but either covered up by drapes or obscured when the patient was positioned. This is particularly likely if the surgical site mark is distant from the nerve-block site. It is not clear whether the wrong-site block was followed by wrong-site surgery but that remains a possibility. On analysis, SALG has concluded that correct implementation of the World Health Organization surgical safety checklist would have prevented wrong- site blocks in the majority of these cases.

A useful poster highlighting a ‘Stop Before You Block’ initiative can be found at: http://www.rcoa.ac.uk/docs/sbyb_posterA4.pdf (cited April 2012).
